# *Helicobacter bilis* Gamma-Glutamyltranspeptidase Enhances Inflammatory Stress Response via Oxidative Stress in Colon Epithelial Cells

**DOI:** 10.1371/journal.pone.0073160

**Published:** 2013-08-23

**Authors:** Sundus Javed, Raquel Mejías-Luque, Behnam Kalali, Christian Bolz, Markus Gerhard

**Affiliations:** Department of Medical Microbiology, Immunology and Hygiene, Technische Universität München, Munich, Germany; University of California, Merced, United States of America

## Abstract

*Helicobacter bilis* (*H. bilis*) infection is associated with cases of inflammatory bowel Disease, thyphlocolitis, hepatitis and cholecystitis. However, little is known about the bacterial virulence determinants or the molecular mechanisms involved. Recently, *H. bilis* γ-glutamyltranspeptidase (HBgGT) was shown to be a virulence factor decreasing host cell viability. Bacterial gGTs play a key role in synthesis and degradation of glutathione and enables the bacteria to utilize extracellular glutamine and glutathione as sources of glutamate. gGT-mediated loss of cell viability has so far been linked to DNA damage via oxidative stress, but the signaling cascades involved herein have not been described. In this study, we identified enhanced ROS production induced by HBgGT as a central factor involved in the activation of the oxidative stress response cascades, which finally activate CREB, AP-1 and NF-κB in *H. bilis* infected colon cancer cells. IL-8, an important pro-inflammatory chemokine that is a common downstream target of these transcription factors, was up-regulated upon *H. bilis* infection in an HBgGT dependent manner. Moreover, the induction of these signaling responses and inflammatory cytokine production in host cells could be linked to HBgGT-mediated glutamine deprivation. This study implicates for the first time HBgGT as an important regulator of signaling cascades regulating inflammation in *H. bilis* infected host epithelial cells that could be responsible for induction of inflammatory disorders by the bacterium.

## Introduction

*Helicobacter bilis* (*H. bilis*), an enterohepatic *Helicobacter* species, is endemic in most mouse facilities and may induce disease in susceptible animals [1]. The bacterium possesses one of the broadest host spectra of the *Helicobacter* genus [2], and *H. bilis* infection has been associated with a higher incidence of typhlocolitis [3,4], Inflammatory Bowel Disease (IBD) [5], hepatitis [6], and cholecystitis [7] in animals. In humans, it has been associated with chronic liver diseases [7,8] and biliary tract and gall bladder cancer [9,10] as well as chronic diarrhea [11] and pyoderma gangrenosum-like ulcers [12]. Chronic inflammation is the underlying cause in many hepatobiliary and gastroenteric disorders, predisposing the tissue to malignant changes. The deregulation of pro-inflammatory chemokines and cytokines such as TNFα, IL-8, IL-6 as well as enzymes such as cyclooxygenase 2 (COX-2) and inducible nitric oxide synthase (iNOS) are frequently implicated in chronic inflammation [13–16]. IL-8 and TNFα up-regulation are a hallmark of IBD [14]. IL-8 functions as a chemoattractant, and is also a potent angiogenic factor [17], which is secreted in large amounts in response to infection and oxidative stress, recruiting inflammatory cells. This in turn results in an additional increase in oxidative stress mediators, making it a key player in localized inflammation [18]. IL-8 is regulated by different transcription factors responding to oxidative stress, including NF-κB, AP-1 and CREB, which directly bind to the IL-8 promoter [19].

NF-κB and CREB transcriptional activities are activated upon infection of bile duct cells with *H. bilis* [20], suggesting an involvement of those transcription factors in the induction of disease upon *H. bilis* infection. Although AP-1 activation has not been described in response to *H. bilis* infections, concomitant activation of AP-1 and NF-κB is often observed during inflammatory diseases, where both transcription factors determine the cytokine gene activation profiles and activity of disease [21]. Moreover, up-regulation of these transcription factors by *H. pylori* is central to the inflammation induced by this bacterium [22].

Although activation of NF-κB and CREB has been described in *H. bilis* infection, the bacterial factors responsible for this induction are unknown. *H. bilis* harbors many virulence factors including urease and cytolethal distending toxin, whose specific function during *H. bilis* infection has not been explored yet [23,24]. Recently, gamma-glutamyl transpeptidase (gGT) has been described as a novel *H. bilis* virulence factor. *H. bilis* genome encodes for two putative gGT sequences, only one of which was found to be functionally active and similar in function to *H. pylori* gGT (HPgGT) in its ability to affect gastric epithelial cell viability [25]. HPgGT represents an important virulence factor of *H. pylori* since it plays an essential role in the colonization of the gastric mucosa and predisposes infected individuals to a higher risk of developing peptic ulcer [26,27]. Furthermore, during *H. pylori* infection, gGT has been described to induce oxidative stress and is one of the bacterial virulence factors responsible for inducing the pro-inflammatory chemokine IL-8 in epithelial cells [27,28]. On the other hand, the effects induced by *H. bilis* gGT (HBgGT) remain largely unknown. Despite the increasing evidence implicating *Helicobacter* gGT in enhanced bacterial virulence, not much effort has gone into elucidating the mechanism of action of this important bacterial enzyme. Thus, gGT-modulated host cell changes leading to inflammation and disease remain mostly elusive. Shibayama et al. proposed that HPgGT may lead to depletion of the antioxidants glutamine and glutathione by gGT enzymatic activity [29]. Interestingly, glutamine depletion has been also implicated in the activation of NF-κB and AP-1 pathways and enhanced IL-8 production by human breast cancer cell line TSE [30]. The presence of gGT in other *Helicobacter* *spp.* underlines its importance in bacterial metabolism and its possible role in inducing inflammatory diseases prevalent in *Helicobacter* infection. Therefore, we aimed at analyzing the effect of HBgGT in colon cancer cells regarding the mechanism involved in induction of transcriptional alterations mediated by oxidative stress signalling as well as possible changes in downstream gene expression.

## Results

### HBgGT up regulates ROS production in colon cancer cells

**Figure 1:**
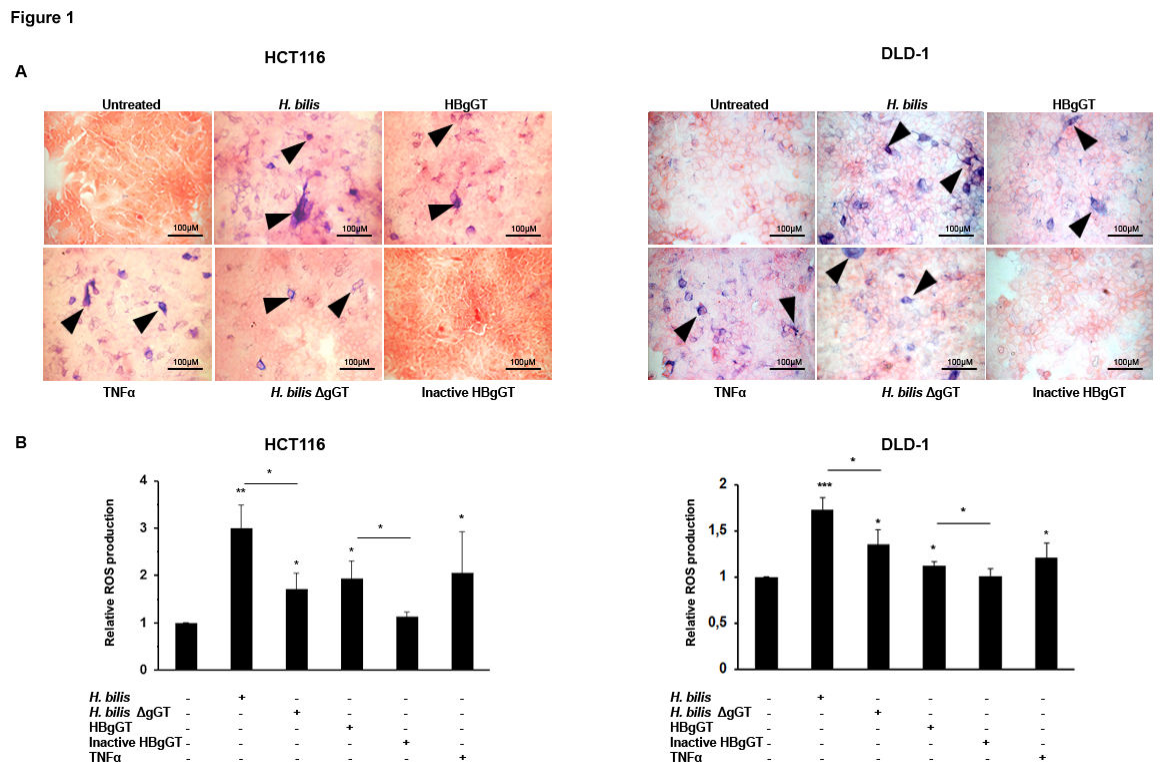
Superoxide production induced by HBgGT in colon cancer cells. A) NBT assay visualization of formazan crystal formation in HCT116 and DLD-1 cells in response to wild type and gGT deficient *H. bilis* infection for 20 hours at MOI 50. Cells were also treated with recombinant HBgGT (5µg/ml) or inactive HBgGT (5µg/ml) for 20 hours. Following treatment, cells were stained for formazan crystals (dark blue) and counterstained with safranin. TNFα (20ng/ml) was used as a positive control. B) Quantification of crystal formation in HCT116 and DLD-1 cell lysates at OD 650. Results are expressed as mean of three independent experiments normalized to the untreated control. *p<0.05, **p<0.005, ***p<0.0005. Asterisks on top of bars indicate significance relative to untreated control; asterisks on bars indicate significance level between indicated conditions.

Generation of reactive oxygen species (ROS) by glutathione hydrolysis has been reported for gGTs from *H. pylori* and *H* *. suis* [31]. We have previously demonstrated similar functional conservation between HPgGT and HBgGT with respect to their immune-evasive potential, which raised the question whether HBgGT might also be involved in the induction of oxidative stress in epithelial cells. We first examined the generation of intracellular superoxide anion radicals (O^2-^) after infection of DLD-1 and HCT116 colon cancer epithelial cell lines with *H. bilis*. Increased accumulation of blue formazan crystal precipitates, which form after superoxide radicals accumulate in the cells under oxidative stress, was observed in cells infected with *H. bilis* when compared to control cells (Figure 1A and 1B). The specific contribution of HBgGT on ROS production was analyzed by infecting the cells with a gGT deficient *H. bilis* strain, which induced markedly diminished superoxide production (Figure 1A and 1B. For characterization of the ΔgGT *H. bilis* see Methods and Figures S1A and S1B), indicating that the presence of gGT in *H. bilis* significantly enhances O^2-^ production from HCT116 (p= 0.0098) and DLD-1 (p=0.024) infected cells.

To assess the ability of HBgGT enzyme alone to induce ROS, colon cancer cells were treated with the recombinant HBgGT or the heat-inactivated protein, defective in catalytic activity (Figures S1C and S1D). HBgGT-treated colon cancer cells exhibited a significantly enhanced formazan precipitate accumulation compared to the untreated control cells (p=0.011 in HCT116 and p=0.0255 in DLD-1 cells), while the inactive enzyme showed no ROS induction compared to untreated control cells (Figure 1A and 1B).

## *H. bilis* Induces gGT-Dependent Oxidative Stress Signaling in Colon Cancer Cells

**Figure 2:**
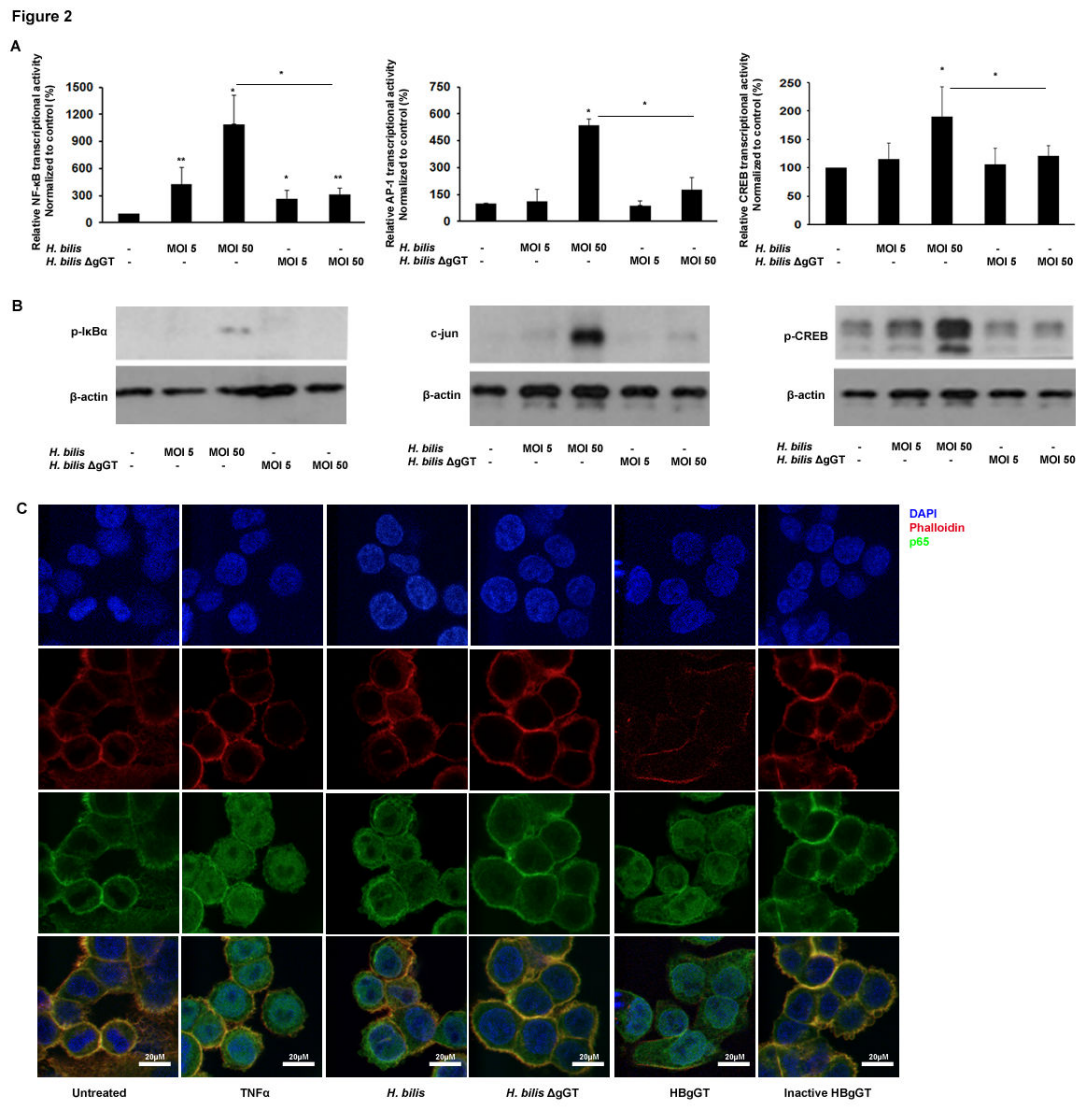
Activation of oxidative stress-associated signaling pathways upon *H. bilis* infection. A) NF-κB, AP-1 and CREB transcriptional activity in gGT proficient and gGT deficient *H. bilis* infected HCT116 colon cancer cells. Transiently transfected HCT116 cells with corresponding luciferase reporter plasmids were co-cultured with *H. bilis* and *H. bilis* ΔgGT at MOI 5 and 50 for 24 hours. Bars represent mean of relative luciferase values to renilla of 3 independent experiments normalized to the untreated control. *p<0.05, **p<0.005. Asterisks on top of bars indicate significance relative to untreated control; asterisks on bars indicate significance level between indicated conditions. B) p-IκBα (40 kDa), c-jun (48kDa) and p-CREB (43 kDa) protein levels detected by western blot. HCT116 cells were lysed after 10 hours of infection at the MOI indicated. β-actin (45 kDa) was used as a loading control. One representative blot of three independent experiments is shown. C) Confocal image of HCT116 cells showing nuclear translocation of NF-κB subunit p65 after 24 hour *H. bilis* infection at MOI 50 or recombinant HBgGT (5µg/ml) treatment. TNFα (20ng/ml) was used to induce nuclear translocation of p65 and the inactive gGT (5µg/ml) was used as control. Actin was stained with phalloidin to allow visualization of total cell area.

Accumulation of ROS has been shown to result in activation of oxidative stress-induced cascades. In order to analyze if HBgGT induces oxidative stress signaling, colon cancer cells were transiently transfected with a luciferase reporter plasmid containing binding sites for transcription factors involved in cellular stress responses including oxidative stress. Specifically, NF-κB, AP-1 and CREB transcriptional activity was tested after *H. bilis* infection at multiplicity of infection (MOI) 5 and 50. Cells were also infected with a gGT deficient bacterium to differentiate between gGT-related effects and those related to other virulence factors. *H. bilis*-infected HCT116 cells exhibited a significant increase in NF-κB (p<0.001), AP-1 (p=0.032) and CREB (p=0.05) transcriptional activities (Figure 2A), and this effect was dose dependent, as an increase in MOI resulted in higher transcriptional activity. Significantly lower levels of NF-κB (p<0.001), AP-1 (p=0.05) and CREB (p=0.048) transcriptional activity were detected when the ΔgGT strain was used at the same MOI. Similar results were obtained with the DLD-1 cell line in the NF-κB and AP-1 reporter assays (Figure S2A); however, high endogenous CREB activation levels compromised the inducibility of CREB in this cell line after treatment (data not shown). To assess CREB levels after *H. bilis* infection, LS174T cells were used instead (Figure S2A), which also demonstrated NF-κB and AP-1 transcriptional activation after *H. bilis* infection (data not shown). Activation of NF-κB, AP-1 and CREB was also confirmed using the recombinant active and heat-inactivated HBgGT enzyme (data not shown).

We next investigated the upstream signaling events leading to activation of the respective transcription factors. Here, we also observed that IκBα phosphorylation, c-jun protein levels and phosphorylation of CREB were induced in a gGT-dependent manner when HCT116 (Figure 2B), DLD-1 and LS174T cells (Figure S2B) were infected with *H. bilis* wild type, but not with the gGT deficient bacterium. Moreover, decreased p65 nuclear translocation could be observed in cells co-cultured with *H. bilis* ΔgGT compared to the wild type as detected by immunofluorescence (Figure 2C).

Taken together, these results indicate that activation of NF-κB, AP-1 and CREB signaling pathways is mostly dependent on gGT activity upon *H. bilis* infection.

**Figure 3:**
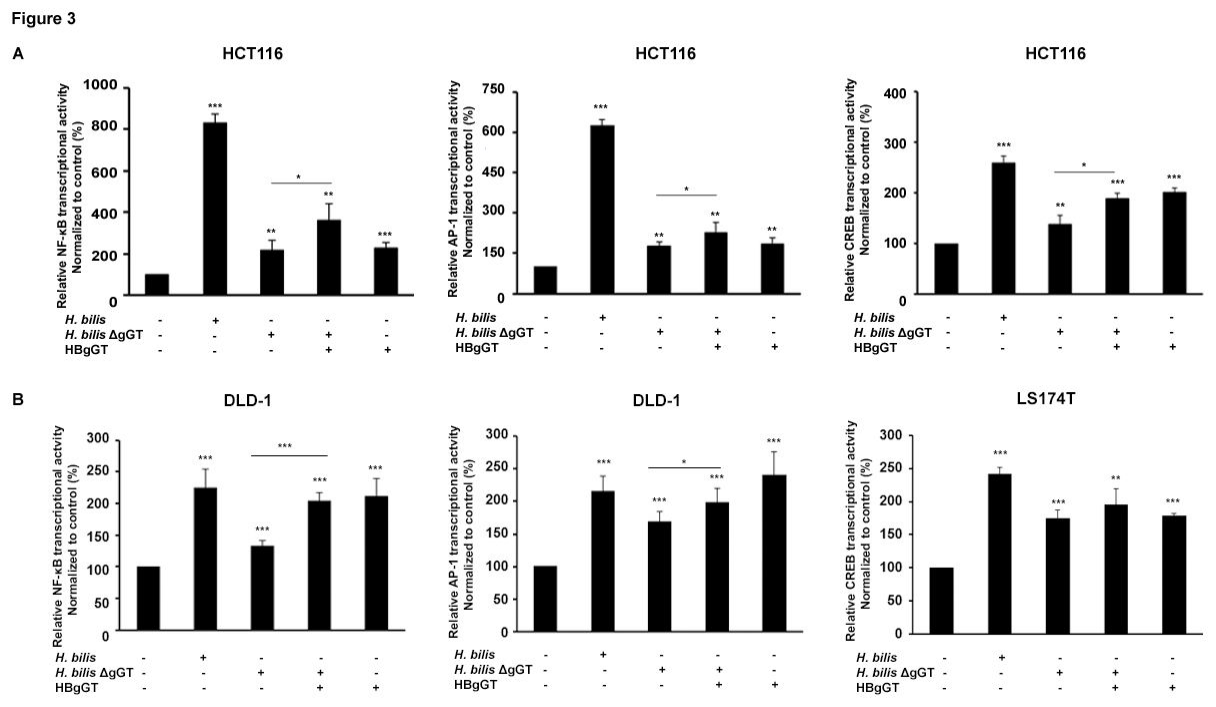
Influence of HBgGT on the activation of the NF-κB, AP-1 and CREB pathways. NF-κB, AP-1 and CREB transcriptional activity in transiently transfected HCT116 (A), and DLD-1 and LS174T (B) cells. Recombinant HBgGT (5µg/ml) was added to *H. bilis* ΔgGT-infected cells and transcriptional activity determined after 24 hours of infection. Results are expressed as mean of relative luciferase activity to renilla of three independent experiments, normalized to the untreated control. *p<0.05, **p<0.005, ***p<0.0005. Asterisks on top of bars indicate significance relative to untreated control; asterisks on bars indicate significance level between indicated conditions.

To further substantiate the gGT involvement in the activation of these pathways, cell co-cultures with the *H. bilis* ΔgGT bacterium were supplemented with the recombinant HBgGT enzyme. Addition of HBgGT to these co-cultures significantly enhanced the transcriptional activation of NF-κB in *H. bilis* ΔgGT-infected HCT116 cells (p=0.028) (Figure 3A) compared to the ones infected with the gGT deficient bacterium only. A similar increase in AP-1 (p=0.05) and CREB (p=0.011) transcriptional activities in *H. bilis* ΔgGT-infected and HBgGT-supplemented cells were observed. However, the increase in the transcriptional activity after HBgGT supplementation of the gGT deficient bacterium did not reach the activation levels observed after infection with wild type *H. bilis*, suggesting the need for continuous gGT secretion from the bacterium during infection to fully activate host signaling cascades. Comparable results were obtained with DLD-1 and LS174T cells (Figure 3B). It must be noted that although the supplementation of recombinant HBgGT was able to enhance CREB transcriptional activation in LS174T cells, the increase was not significant. This could be attributed to the differential inducibility and sensitivity of the different cell lines to treatment. As observed previously, these transcriptional changes were accompanied by increased phosphorylation of IκBα and CREB, as well as enhanced levels of c-jun after HBgGT addition to *H. bilis* ΔgGT (data not shown) supporting an involvement of HBgGT in the activation of these signaling pathways.

### HBgGT enhances H. bilis-induced IL-8 production from epithelial cells

**Figure 4:**
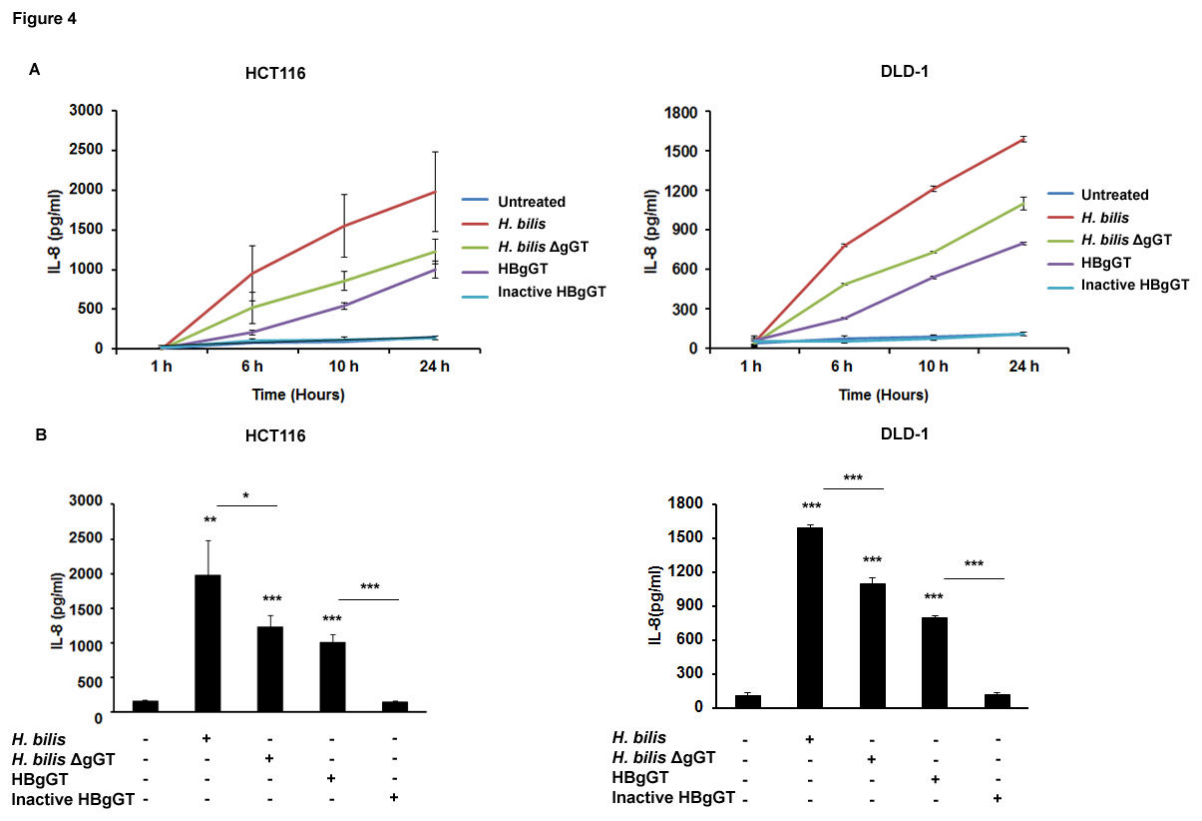
Influence of HBgGT on IL-8 secretion by colon cancer cells. A) IL-8 secretion determined by ELISA in cell culture supernatants of HCT116 and DLD-1 cells treated with recombinant HBgGT (5µg/ml) or infected with *H. bilis* or *H. bilis* ΔgGT (MOI 50) for different time points. Mean values of three independent experiments are shown. B) IL-8 levels after 24 hour of treatment secreted by HCT116 and DLD-1 cells upon HBgGT treatment or *H. bilis* infection. Results are expressed as mean of three independent experiments. *p<0.05 **p<0.005, ***p<0.0005. Asterisks on top of bars indicate significance relative to untreated control; asterisks on bars indicate significance level between indicated conditions.

IL-8 plays an important role as a mediator of the innate immune response to different bacteria including some *Helicobacter* species. Since transcriptional activation of NF-κB, AP-1 and CREB was induced by HBgGT and binding sites for these three transcription factors have been identified in the IL-8 promoter, we sought to investigate whether *H. bilis* infection was able to trigger IL-8 secretion from epithelial cells in a gGT dependent manner. Therefore, IL-8 content in supernatants from colon cancer cells infected with *H. bilis* or treated with recombinant gGT was measured by ELISA. Exposure of cells to *H. bilis* bacteria or recombinant HBgGT significantly induced IL-8 secretion in HCT116 (p<0.001) and DLD-1 (p<0.001) cells in contrast to the cells treated with the inactive gGT (Figure 4A and 4B). While the gGT deficient bacterium was also able to induce IL-8 secretion from HCT116 (p= 0,033) and DLD-1 (p<0.001) cells, the IL-8 release was significantly lower compared to that induced by gGT proficient bacteria. This suggests that although gGT significantly enhances the IL-8 production from epithelial cells, it is not the only bacterial factor contributing to IL-8 production from *H. bilis* infected cells.

### Regulation of gGT-mediated transcriptional activation involves glutamine deprivation

**Figure 5:**
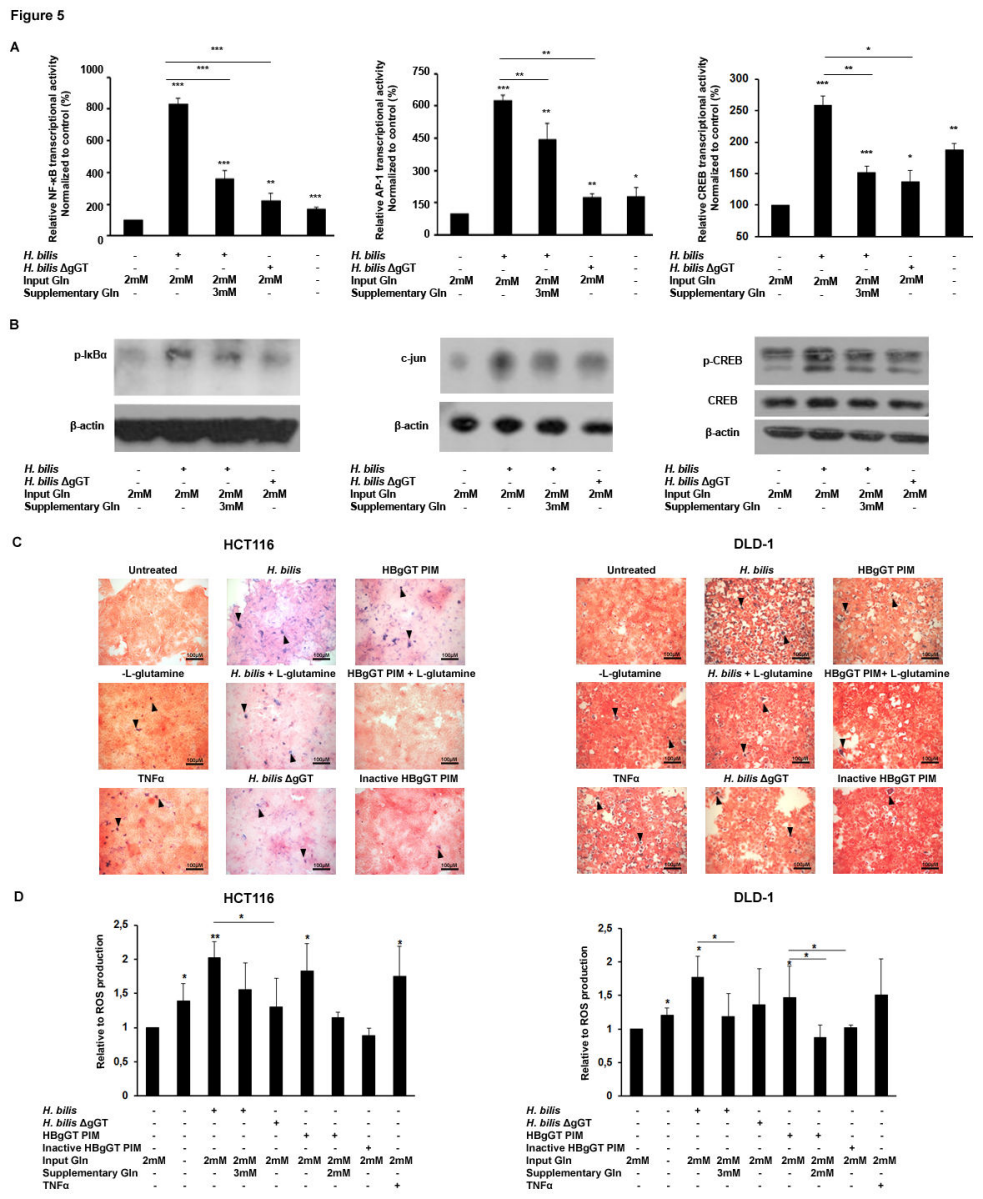
Effect of glutamine supplementation on the activation of NF- κB, AP-1 and CREB pathways after *H. bilis* infection. A) NF-κB, AP-1 and CREB transcriptional activity. Transiently transfected HCT116 cells were infected with *H. bilis* (MOI 50). Infected cells were supplemented with 3mM L-glutamine (Supplementary Gln) in addition to the 2mM present in the culture medium (Input Gln) for 24 hours where indicated. Untreated cells cultured in 2mM L-glutamine in culture medium served as the untreated control. L-glutamine free medium was used to starve the cells of glutamine for the same time period. Results are expressed as mean of relative luciferase activity to renilla of three independent experiments, normalized to the untreated control. *p<0.05, **p<0.005, ***p<0.0005. Asterisks on top of bars indicate significance relative to untreated control; asterisks on bars indicate significance level between indicated conditions. B) p-IκBα, c-Jun and p-CREB protein levels analyzed by western blot in HCT116 after 10 hours *H. bilis* infection (MOI 50). Where indicated regular cell culture medium containing 2mM L-glutamine (Input Gln) was supplemented with additional 3mM L-glutamine (Supplementary Gln). β-actin was used as a loading control. One representative blot from three independent experiments is shown. C) ROS production was determined by NBT assay in HCT116 and DLD-1 cells after L-glutamine supplementation of cells infected with *H. bilis* (MOI 50) or treated with HBgGT PIM (pre-incubated medium with 5µg of HBgGT/ml of culture medium) for 20 hours. L-glutamine free medium was used to starve the cells of glutamine. TNFα (20µg/ml) was used as a positive control. D) Quantification of superoxide production in similarly treated HCT116 and DLD-1 cell lysates at OD 650. Results are expressed as mean of three independent experiments, normalized to the untreated control. *p<0.05, **p<0.005, ***p<0.0005. Asterisks on top of bars indicate significance relative to untreated control; asterisks on bars indicate significance level between indicated conditions.

We next investigated the mechanism by which HBgGT could induce ROS production and activation of the NF-κB, AP-1 and CREB signaling cascades. Since gGT is able to hydrolyze glutamine (and to a lesser extent glutathione) as a substrate to be utilized in bacterial glutamate synthesis, depletion of extracellular glutamine due to HBgGT enzymatic activity might be an important factor impairing the redox balance of the host cell, thereby rendering it prone to ROS generation and activation of related signaling pathways. Indeed, we could confirm that glutamine deprivation was able to trigger transcriptional activities of NF-κB (p<0.001), AP-1 (p=0.027) and CREB (p<0.001) in HCT116 cells cultured in glutamine free medium for 24 hours (Figure 5A). Moreover, the addition of glutamine to *H. bilis*-infected cells was able to significantly lower the transcriptional activities of NF-κB (p<0.001) as well as of AP-1 (p=0.016) and CREB (p=0.001), while infection under glutamine-depleted conditions led to cell death (data not shown). Optimal amounts of supplementary L-glutamine to be added for successful protection against stress response activation were pre-determined using a dose response curve to IL-8 (Figure S4). It must be noted that glutamine was not able to completely reverse these inductions to the levels observed with the deletion mutant. Such reduced activation of the NF-κB, AP-1 and CREB pathways was accompanied by a significant decrease in IκBα and CREB phosphorylation and reduced total c-jun levels after addition of glutamine to infected cells compared to the infected cells alone (Figure 5B). A similar effect was observed in DLD-1 and LS174T cells as shown in Figures S3A and S3B. These results indicate that HBgGT-induced transcriptional changes in host cells partially occur through glutamine deprivation.

Furthermore, glutamine deprivation by itself induced a significant increase in the levels of intracellular superoxide anion radicals in colon cancer cells (Figure 5C and Figure 5D). In contrast, glutamine supplementation of *H. bilis*-infected DLD-1 cells significantly reduced ROS production (p= 0.046), indicating that HBgGT enhances oxidative stress partly by glutamine depletion. We therefore conclude that HBgGT enzymatic activity-dependent glutamine depletion is the initial step in inducing ROS and oxidative stress response. This finally leads to IL-8 secretion via activation of NF-κB, AP-1 and CREB.

#### Glutamine supplementation lowers IL-8 levels secreted by *H. bilis* infected cells

As glutamine supplementation was able to effectively reduce the activation levels of the oxidative stress response cascades studied, we next examined if glutamine deprivation could also decrease cellular IL-8 levels secreted in response to *H. bilis.*

**Figure 6:**
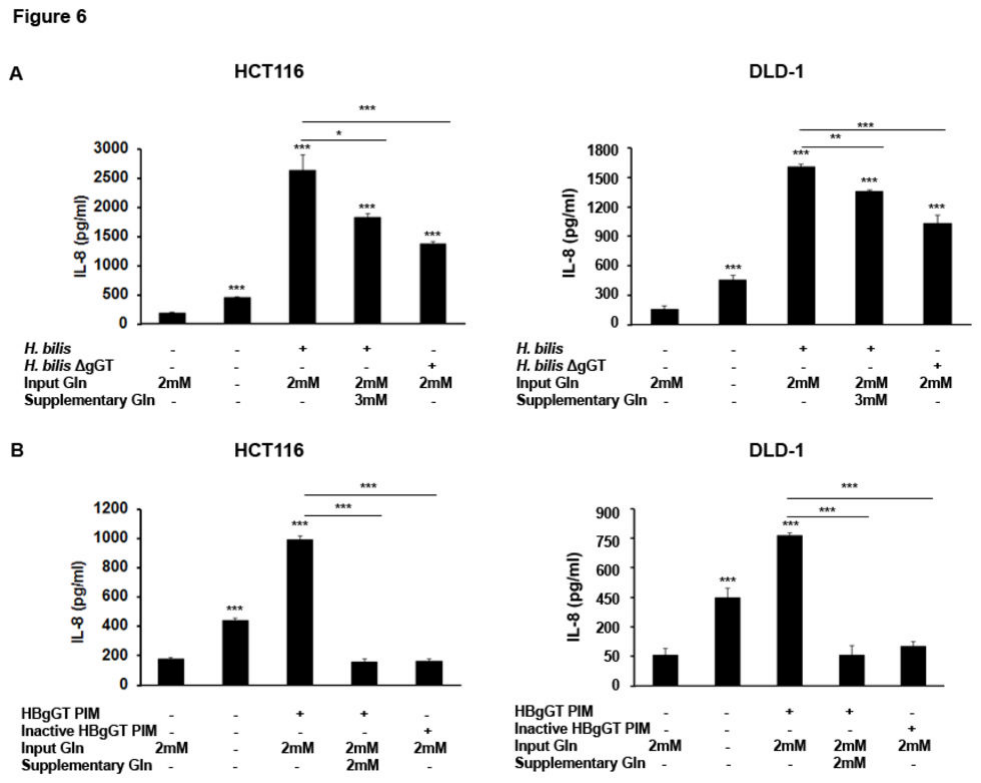
Influence of glutamine supplementation on IL-8 secretion by *H. bilis* co-cultures and HBgGT-treated cells. A) IL-8 production by HCT116 and DLD-1 cells. *H. bilis* (MOI 50) infected cells were supplemented with 3mM L-glutamine (Gln). Supernatants of 24 hour treated cells were collected and IL-8 secretion determined by ELISA. Results are expressed as mean of three independent experiments. *p<0.05, **p<0.005, ***p<0.0005. B) IL-8 levels secreted by HCT116 and DLD cells after glutamine supplementation (Gln 2mM, where indicated) of HBgGT PIM (pre-incubated medium, 5µg HBgGT/ml of cell culture medium). Heat-inactivated HBgGT PIM (inactive HBgGT pre-incubated at 5µg/ml of culture medium) was used as an enzymatically inactive control. Results are expressed as mean of three independent experiments. *p<0.05, **p<0.005, ***p<0.0005.

First, we observed that starvation of cells from extracellular glutamine sources alone was able to induce the secretion of IL-8 by HCT116 (p<0.001) and DLD-1 (p<0.001) colon cancer cells (Figure 6A and Figure 6B), although at lower levels compared to *H. bilis* infection. Furthermore, glutamine supplementation of *H. bilis*-infected cells significantly lowered the IL-8 levels secreted from HCT116 (p<0.001) and DLD-1 (p< 0.001) cells (Figure 6A). To further confirm the effect of glutamine deprivation on gGT-mediated IL-8 induction, cells were treated with HBgGT pre-incubated medium for 24 hours, which resulted in comparable IL-8 secretion to the levels observed after treatment with the recombinant protein. Interestingly, the IL-8 secretion induced by HBgGT pre-incubated medium could be restored to basal levels when the pre-incubated medium was supplemented with 2mM glutamine in HCT116 (p<0.001) and DLD-1 (p<0.001) (Figure 6B). These data support our hypothesis that glutamine deprivation plays a key role in HBgGT-modulated host cell changes.

## Discussion

*H. bilis* infection has been linked to development of IBD, colitis as well as biliary tract and gall bladder cancers in various host species [1,3,4,7,32]. However, the molecular mechanisms underlying the infection in the host cell leading to disease as well as the bacterial virulence factors involved still remain elusive.

In a previous study, we identified HBgGT as an important virulence determinant affecting host cell proliferation, and demonstrated that HBgGT exhibits similar affinity to L-glutamine as HPgGT [25]. gGT is a threonine N-terminal nucleophile hydrolase which catalyzes the transpeptidation and hydrolysis of the γ-glutamyl moiety of glutathione and related compounds. The reaction is accompanied by generation of ROS as well as ammonia [33]. Glutamine hydrolysis by gGT deprives cells of its antioxidant properties and leads to ROS generation. In the presence of molecular oxygen and iron or copper ions, a number of antioxidants paradoxically generate ROS leading to free radical damage of nucleic acids and oxidative modification of lipids and proteins. In fact, human gGT (HsgGT) is able to generate ROS in the presence of glutathione and transferrin as an iron source [34], while ROS induction by bacterial gGTs from *H. pylori* and *H* *. suis* induces host epithelial cell necrosis in presence of glutathione [31]. Furthermore, ROS and reactive nitrogen species generated due to gGT activity are mediators of cell signaling in epithelial cells.

In the present study we observed increased levels of superoxide production from *H. bilis*-infected cells, which partly depended on the presence of gGT. Molecular mechanisms of ROS action are only partially understood; it is hypothesized that ROS may lead to oxidation of disulfide groups in redox sensitive proteins with highly conserved cysteine residues that may cause structural changes leading to the exposure of active sites and subsequent activation. Such molecular targets include transcription factors NF-κB and AP-1, signaling molecules such as Ras/Rac or JNK and protein tyrosine phosphatases [35–37]. Also, Felty and Roy observed that stimulation of redox sensor kinase A-Raf, AKT or PKC, activates transcription factors NF-κB, CREB, or AP-1 [37]. We observed that *H. bilis* infection of colon cancer cells indeed activated the oxidative stress-associated signalling pathways NF-κB, AP-1, and CREB. Induction of these and other transcription factors has been reported in response to *Helicobacter* infection in epithelial cells [38–44], while *H. bilis* infection in bile duct cells has been shown to influence CRE transcriptional activity [20]. However, in our study, such activation was significantly enhanced when the bacteria were gGT proficient, suggesting that HBgGT plays an important role in the regulation of these signalling cascades during *H. bilis* infection. We noted that cells treated with the recombinant HBgGT show a weaker cellular stress response compared with the *H. bilis* wild type-infected cells, possibly because the bacteria constantly produce the protein, while the recombinant protein was only added once. The residual activation of the pathways in the host cells when infected with the gGT knockout bacterium indicates that other virulence factors, such as the cytolethal distending toxin, peptidoglycan or LPS, may additionally contribute to the effects observed. This assumption is supported by studies in several helicobacters also showing gGT independent NF-κB signalling i.e. for *H. pylori*, *H* *. muridarum* and recently *H. bilis* [20,22,45].

ROS-induced activation of NF-κB, AP-1 and CREB may in turn play a major role in inflammation. Oxidative stress induced by H_2_O_2_ and TNF-α increase the activation of AP-1 and NF-κB, which lead to IL-8 expression [30,46,47]. IL-8 is associated with inflammation and is one of the main mediators in the immune response particularly against *Helicobacter* spp. infections. We observed increased secretion of IL-8 after HBgGT treatment of colon cancer cells. Furthermore, *H. bilis* was able to induce higher levels of IL-8 expression by colon epithelial cells when expressing gGT, pointing to the fact that HBgGT significantly contributes to *H. bilis*-induced host cell inflammatory response. Having established gGT as an important virulence factor in the *Helicobacter* arsenal, we sought to determine the mechanism behind the induction of oxidant stress induced by HBgGT, and could link it to its intrinsic enzymatic activity.

**Figure 7:**
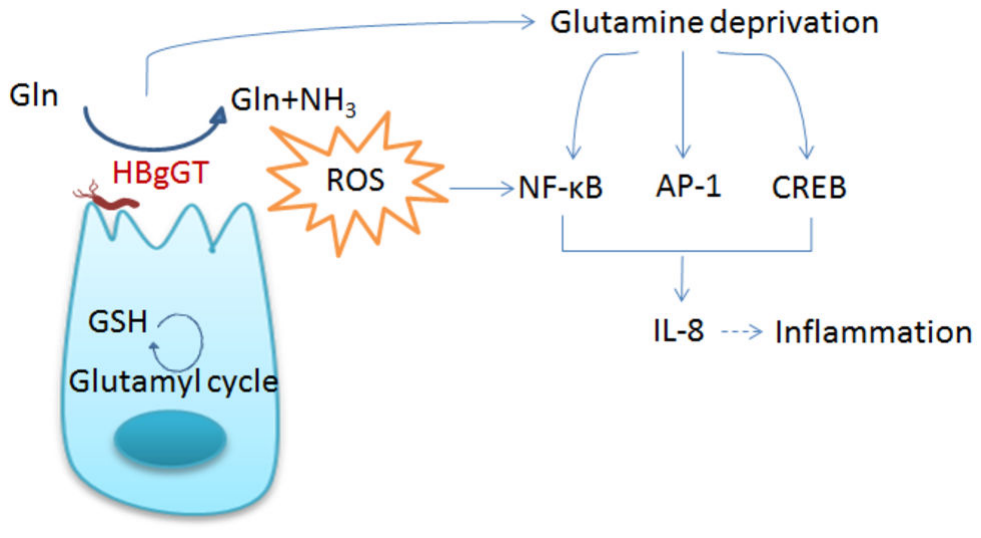
Model of host cell response modulated by HBgGT. Glutamine deprivation, exhaustive consumption of glutathione, and subsequent generation of free radicals by *H. bilis* gGT induce several oxidative stress response cascades in host cells, cumulating in IL-8 secretion.

One of the main physiological functions of *Helicobacter* gGT is to enable the bacteria to utilize extracellular glutamine and glutathione as sources of glutamate, and, indeed, we previously showed that HBgGT is able to hydrolyze glutamine [25]. In mammalian cells glutathione is a ubiquitous substance present in the cytosol in mM quantities [48], and glutamine is essential for maintaining homeostasis and normal integrity of intestinal mucosa [49–51]. Glutamine and glutathione function as anti-oxidants by detoxification of oxidizing substances [49,51], and glutamine depletion leads to an impaired redox balance, triggering a whole cascade of oxidative stress response elements [30,35,52,53]. To analyze the influence of gGT-dependent glutamine depletion on the cellular stress response, glutamine supplementation experiments were performed. We observed that glutamine supplementation of HBgGT-treated or *H. bilis*-infected cells was able to diminish gGT-induced activation of NF-κB, AP-1, CREB as well as ROS and IL-8 production. Glutamine protects epithelial tight junctions as well as serves as a precursor for glutathione synthesis [54–56]. Depletion of cellular glutamine resources not only affects cell growth and viability but also limits intracellular glutathione reserves, an important anti-oxidant, thereby compromising host cell protection against infection and oxidant stress [57,58]. At this point, we cannot rule out that the glutamine depletion observed here might induce changes in intracellular glutathione levels, since gGT can also hydrolyze glutathione. However, data in the literature on the effects of glutathione depletion on oxidative stress signaling and specifically NF-κB and AP1 regulation are conflicting, because glutathione depletion has also been reported to down regulate NF-κB and AP1 responses [59,60]. Our observations are in agreement with previous studies showing that glutamine depletion from cellular sources can cause increased activation of the NF-κB and AP-1 signaling pathways leading to an augmented expression of IL-8 [30,52,60], and provide with a novel mechanism by which HBgGT modulates host cell response to *H. bilis* infection (summarized in Figure 7).

The protective role of glutamine against host cell transcriptional alterations and IL-8 production in the context of *H. bilis* infection observed in our study is in accordance with reports describing the protective effect of glutamine against intestinal inflammation [50,61–65]. Mechanistically, deprivation of glutamine causes increased activation of the NF-κB pathway, leading to enhanced sensitization of the cells to LPS induced IL-8 production [30,52] which explains the higher IL-8 levels observed in *H. bilis* infected cells compared to the gGT mutant bacterium, where gGT may exhaust the cellular glutamine supply. Increased NF-κB (p65/p50) and AP-1 (Fra-1/c-Jun, JunD) DNA-binding activities were found in response to glutamine deprivation, leading to an increased IL-8 expression [30]. *In vivo*, glutamine supplementation has been shown to improve the outcome of experimentally induced colitis in rats by attenuating cytokine- induced inducible nitric oxide production and nuclear translocation of nuclear factor-κB p65 subunit [61]. Since glutamine supplementation in *H. bilis* infected cells was also able to decrease activation of the NF-κB, AP-1 and CREB pathways as well as lower IL-8 production in the colon cancer cells used in our study, glutamine administration could be considered as a protective therapeutic approach against *H. bilis* induced IBD and colitis.

The results presented here indicate a significant role of HBgGT in host epithelial cell responses towards *H. bilis* infection, and provides possible underlying molecular mechanisms. Our data indicate that the HBgGT mediated glutamine depletion leads to ROS-mediated activation of NF-κB, AP-1 and CREB and subsequent IL-8 secretion upon *H. bilis* infection of colon cancer cells, adding new insights into *H. bilis*-induced pathogenesis. However, further studies are required to understand the underlying bacterium-host dynamics during infection. Moreover, *in vivo* validation will help to understand the role of gGT in *H. bilis*-induced inflammation and pathogenesis.

## Materials and Methods

### Cell cultures

Colon cancer cell lines HCT116 (CCL-247), DLD-1 (CCL-221) and LS174T (CCL-188) were purchased from ATCC and maintained in DMEM (GIBCO, Invitrogen, Carlsbad CA, USA) containing 2mM L-glutamine (GIBCO, Invitrogen, CA, USA) (except where indicated) supplemented with 10% FBS (GIBCO, Invitrogen, CA, USA) and 1% Penicillin/ Streptomycin (GIBCO, Invitrogen, CA, USA). All cell lines were maintained in an incubator at 37°C with 5% CO_2_ and 100% humidity.

### Bacterial Culture

Bacterial cultures were grown on Wilkins-Chalgren (WC)-blood agar plates containing DENT supplement and kept at 37°C in a microaerobic atmosphere (10% CO_2_; 5% O_2_). *H. bilis* (ATCC 43879) could be maintained with minimum viability loss for up to 3 days in culture after which the bacteria were sub-cultured onto fresh agar plates. Bacterial cells were only sub-cultured up to 3 times to minimize genotypic and/or phenotypic changes*. H. bilis* gGT deletion mutant was kindly provided by M. Rossi [25]. Deletion of gGT was confirmed by PCR using gGT specific primer amplification of genomic DNA and enzymatic activity assay, performed with the bacterial culture supernatants (Figures S1A and S1B). No differences in growth were observed between the wild type and gGT-deficient bacteria.

### Antibodies and recombinant proteins

p-IκBα, c-jun, p-CREB and CREB antibodies were purchased from Cell Signaling, (Beverly, Massachusetts, USA). Anti-p65 antibody was purchased from Santa Cruz (California, USA) and anti-β-actin was obtained from Sigma-Aldrich (Missouri, USA). Peroxidase labeled anti-mouse and anti-rabbit IgG antibodies were obtained from Promega (Mannheim, Germany), fluorescence labeled Alexa Flour 488 rabbit anti-mouse antibody was purchased from Invitrogen (California, USA) and phalloidin was from Dyomics (Jena, Germany). Recombinant TNFα was obtained from Preprotech (Hamburg, Germany). The recombinant HBgGT protein was purified according to established protocols [25]. The protein was inactivated by heating at 95^°^C for 5 minutes. Pre-incubated medium was generated by incubating 5µg/ml of recombinant HBgGT in cell culture medium for 24 hours, prior to heat inactivation of the enzyme.

### Cell-Bacterial co-culture

*H. bilis* cultured WC-DENT agar plates were incubated for 1 day before the bacteria were used for inoculation. Bacteria were suspended in DMEM and adjusted OD to 1.0 (2x10^8^ CFUs/ml). 80% confluent cells were counted (after trypsinization) and bacteria added to an MOI of 5 or 50, as indicated.

### Superoxide anion quantification

Production of intracellular superoxide anion (O_2_^-^) was measured using the NBT assay, whereby Nitroblue tetrazolium salt (Sigma-Aldrich, Missouri, USA) is reduced by O_2_^-^ resulting in accumulation of dark blue formazan crystals in the cells.

Cells were grown in 24 well plates and treated for 20 hours. Medium was removed after the treatment period and cells were washed once with DMEM. 250µl of 0.2% NBT solution was added per well and the cells incubated for an additional 1 hour. Photometrical quantiﬁcation was done by fixing the cells after NBT incubation with 100% methanol for 15 minutes. The cells were washed twice with 70% methanol and left to dry overnight in a fume hood. Cells were lysed by addition of 62.5µl per well of KOH and 75µl per well DMSO and homogenized by shaking the plate. 100µl of the lysate was transferred to a 96 well plate. The experiment was performed in duplicates and color development was recorded at 650 nm.

For visualization of formazan crytals, cells were grown on coverslips and treated for the same time. 250µl of 0.2% NBT was added per well after washing with DMEM and incubated for 1 hour before ﬁxation. Cells were fixed with 4% PFA for 15 minutes and nuclei were counterstained for 30 seconds by 0.1% safranin O (Sigma-Aldrich, Missouri, USA) in PBS. Cells were microscopically analyzed using the AxioVert 40 Microscope (Zeiss) and images acquired via the Axio Vision Rel. 4.4 software (Zeiss).

### Luciferase reporter assays

Transient transfections were carried out in colon cancer cells with lipofectamine (Invitrogen, CA, USA) by using 750ng of the luciferase reporter plasmids containing three binding sites for NF-κB, AP-1 or CREB. NF-κB and AP-1 luciferase reporter plasmid were kindly provided by Florian Greten and Roland M. Schmid, respectively. CREB plasmid was constructed by Behnam Kalali. Cells were cotransfected with 20ng of simian virus 40-Renilla luciferase plasmid (Promega, Mannheim, Germany) to account for differences in transfection efficiency. The expression of firefly and renilla luciferases was measured using the Dual Luciferase Reporter Assay System (Promega, Mannheim, Germany) after a certain treatment period, according to the manufacturer’s instructions. The experiment was performed in duplicates and the relative luciferase activity was defined as luciferase reporter plasmid activity normalized to renilla luciferase values.

### Immunofluorescence

Cells (1x10^4^ per well) were grown in 8 well chamber slides. Cells were serum starved for 20 hours after attachment. Culture medium was replenished with 10% serum before treatment with recombinant HBgGT or infection with *H. bilis*. Following treatment for 20 hours, the cells were washed once with PBS and fixed with 2.6% PFA in 75mM sodium phosphate pH 7.4 for 15 minutes. Following washing for 3 times with PBS chamber slides were transferred to a humidified chamber and permeabilized in 0.05% Triton-X100, 3% BSA, 1% saponin PBS for 15 min at room temperature. Permeabilization buffer was removed and cells incubated with p65 primary antibody (diluted 1:300 in 3% BSA, 1% saponin, PBS) overnight at 4°C. Incubation with fluorescence labeled Alexa Flour 488 rabbit anti-mouse secondary antibody (diluted 1:750 in 3% BSA, 1% saponin PBS) was for 4 hours at 4°C. After incubation, antibody solution was removed and cells were washed 3 times for 5 minutes with 1% saponin PBS. Actin filaments were subsequently stained with phalloidin, diluted 1:10 in PBS for 30 minutes. Cells were washed 3 times with PBS before mounting with Vectashield containing DAPI (Vector laboratories, CA, USA).

Samples were visualized with a confocal florescence microscope. Images were acquired with LAF-AS (Leica, Wetzler, Germany) arranged and assembled with the ImageJ software (National Institutes of Health, MD, USA) and Photoshop CS (Adobe Systems, CA, USA).

### Western blot

For analysis of the phosphorylated or total protein levels of IκBα, c-jun and CREB; stimulated cells at different time points were rinsed twice with PBS and then lysed in SDS lysis buffer (0.25M Tris-HCl, pH 6.8, containing 20mM DTT, 6% SDS, 10% glycerol and 2.5mg bromophenol blue). Cell lysates were sonicated and boiled before protein separation on a 10% SDS–PAGE gel. After electrophoresis, proteins were transferred onto nitrocellulose membranes (Whatman/GE Healthcare, Freiburg, Germany), which were blocked in 5% skim milk for 1 hour at room temperature and incubated with primary antibodies following manufacturer’s instructions. After washing, secondary antibodies were incubated for 1 hour at RT and finally visualized by ECL Western Blotting Detection reagents. β-actin was used as a loading control.

### ELISA

Cells were grown in 12 well tissue culture plates. Following treatment, supernatants from the cells were collected and cleared by centrifugation at 13000 rpm. IL-8 ELISA was performed in duplicates according to the manufacturer’s instructions using the IL-8 kit from eBioscience, (CA, USA).

### Glutamine supplementation experiments

To elucidate protective effects of L-glutamine supplementation, cells were seeded as described earlier with an initial input concentration of 2mM L-glutamine in the culture medium. Cells were serum starved for 24 hours before serum free medium was replaced with DMEM containing 10% FBS and 2mM input L-glutamine. Cells were then either infected as described earlier or treated with HBgGT pre-incubated medium (PIM), (described in the recombinant protein section). After infection and/or treatment of cells, supplementary amounts of L-glutamine (Supplementary Gln) were added to the culture medium, in addition to that present before infection (2mM input L-glutamine). Cells cultured in glutamine free medium (no input Gln) were also used as a comparative control in addition to the ones cultured in medium containing 2mM input glutamine.

### Statistics

Mean values and SEMs were calculated from at least three independent experiments. Statistical analysis was performed using the Student’s T-Test. Statistical significance was established when p value was ≤ 0.05.

## Supporting Information

Figure S1 A) gGT screening PCR was performed to confirm insertion of a chloramphenicol resistance cassette into the gGT sequence leading to gene disruption in the *H. bilis* Δggt strain. *H. bilis* wild type bacterium was used as a control.
B) gGT activity assay measured in supernatants of *H. bilis* wild type and ΔgGT bacteria.
C) Recombinant HBgGT protein as well as the heat inactivated HBgGT protein were analysed via SDS PAGE to determine purity.
D) gGT activity assay of the recombinant HBgGT and the inactive enzyme after heat inactivation at 95°C for 5 minutes.
(TIF)

Figure S2 A) NF-κB and AP-1 transcriptional activity in infected DLD-1 cells as well as CREB transcriptional activity in LS174T cells. Transiently transfected DLD-1 and LS174T cells were co-cultured with *H. bilis* and *H. bilis* Δggt at MOI 5 and 50 for 24 hours. Bars represent mean of relative luciferase values to renilla normalized to the untreated control of 3 independent experiments. *p<0.05, ** p<0.005, ***p<0.0005. Asterisks on top of bars indicate significance relative to untreated control; asterisks on bars indicate significance level between indicated conditions.
B) Western blot analysis of p-IκBα and c-jun protein levels in DLD-1 cells and p-CREB expression in LS174T after 10 hours *H. bilis* infection. TNFα (20ng/ml), forskolin (10µM) and PMA (0.5µg/ml) were used as positive controls. β-actin was used as a loading control. One representative blot is shown.
(TIF)

Figure S3 A) NF-κB, AP-1 and CREB transcriptional activity in DLD-1 and LS174T cells after glutamine supplementation of *H. bilis* (MOI 50) infected cells. Cells were transiently transfected with a luciferase reporter plasmid and infected with *H. bilis* at an MOI of 50. 3mM of L-glutamine (Supplementary Gln) was added in addition to the 2mM already present in the culture medium. *H. bilis* Δggt infected cells, at MOI of 50 were used as a control. L-glutamine free medium was used to starve the cells of glutamine. Results are expressed as mean of relative luciferase activity to renilla of three independent experiments, normalized to the untreated control. *p<0.05,**p<0.005, ***p<0.0005. Asterisks on top of bars indicate significance relative to untreated control; asterisk on bars indicate significance level between indicated conditions.
B) Western blot analysis of p-IκBα and c-Jun protein levels after glutamine supplementation of *H. bilis* (MOI 50) infected DLD-1 cells after 10 hours of treatment. CREB phosphorylation was investigated in LS174T cells after 10 hours of treatment. One representative blot is shown.
(TIF)

Figure S4 A) IL-8 production in HCT116 cell culture supernatants determined by ELISA in response to increasing supplementary L-glutamine concentrations after 24 hours of *H. bilis* (MOI 50) infection. Results from two independent experiments conducted in duplicates are shown.
B) IL-8 levels after glutamine supplementation of HBgGT PIM treated HCT116 cells at increasing dosage. Supernatants of 24 hour treated cells were collected and IL-8 secretion determined by ELISA. Data from two independent experiments conducted in duplicates are shown.
(TIF)
