# Supplementary figures and images for: Helicobacter bilis Gamma-Glutamyltranspeptidase Enhances Inflammatory Stress Response via Oxidative Stress in Colon Epithelial Cells
Source: PLoS One. 2013 Aug 23;8(8):e73160. doi: 10.1371/journal.pone.0073160 (PMC3751837; doi:10.1371/journal.pone.0073160)

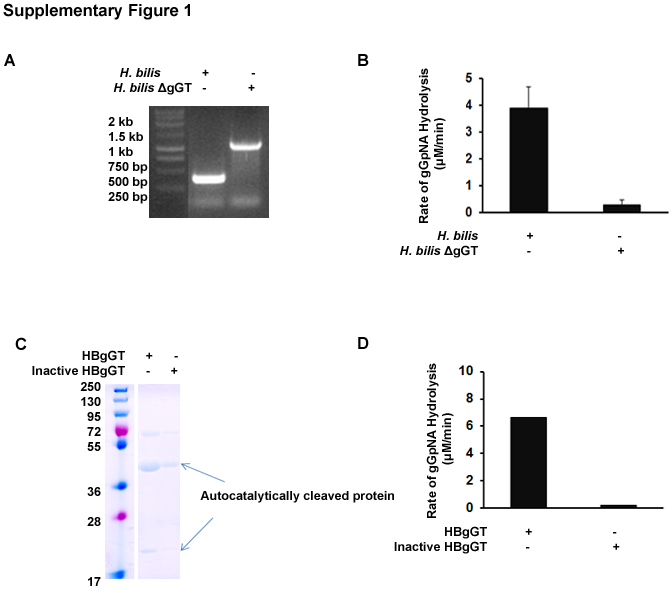

Supplement: Figure S1 — A) gGT screening PCR was performed to confirm insertion of a chloramphenicol resistance cassette into the gGT sequence leading to gene disruption in the H. bilis Δggt strain. H. bilis wild type bacterium was used as a control. B) gGT activity assay measured in supernatants of H. bilis wild type and ΔgGT bacteria. C) Recombinant HBgGT protein as well as the heat inactivated HBgGT protein were analysed via SDS PAGE to determine purity. D) gGT activity assay of the recombinant HBgGT and the inactive enzyme after heat inactivation at 95°C for 5 minutes. (TIF) [file pone.0073160.s001.tif]

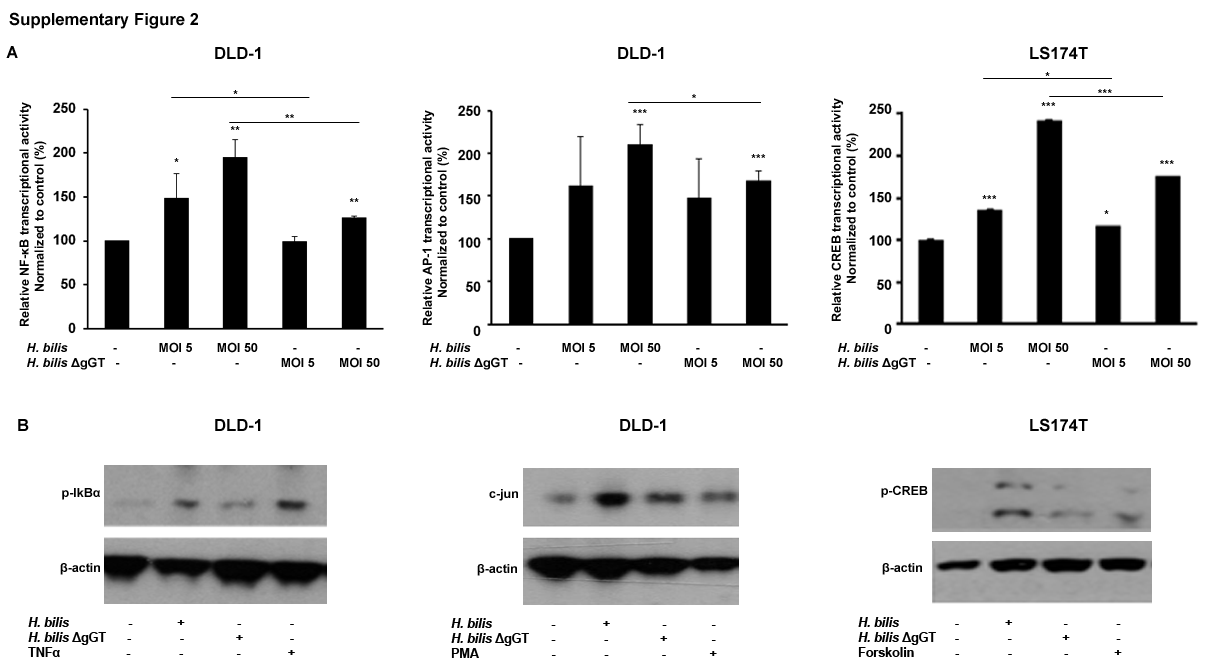

Supplement: Figure S2 — A) NF-κB and AP-1 transcriptional activity in infected DLD-1 cells as well as CREB transcriptional activity in LS174T cells. Transiently transfected DLD-1 and LS174T cells were co-cultured with H. bilis and H. bilis Δggt at MOI 5 and 50 for 24 hours. Bars represent mean of relative luciferase values to renilla normalized to the untreated control of 3 independent experiments. *p<0.05, ** p<0.005, ***p<0.0005. Asterisks on top of bars indicate significance relative to untreated control; asterisks on bars indicate significance level between indicated conditions. B) Western blot analysis of p-IκBα and c-jun protein levels in DLD-1 cells and p-CREB expression in LS174T after 10 hours H. bilis infection. TNFα (20ng/ml), forskolin (10µM) and PMA (0.5µg/ml) were used as positive controls. β-actin was used as a loading control. One representative blot is shown. (TIF) [file pone.0073160.s002.tif]

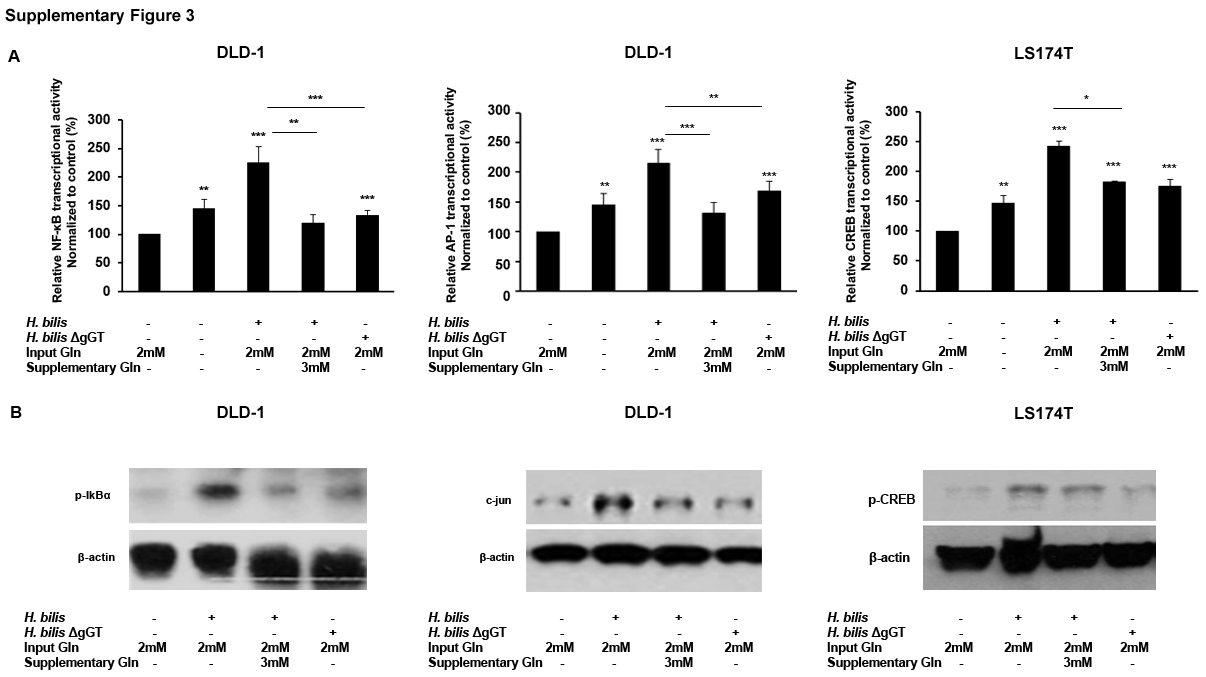

Supplement: Figure S3 — A) NF-κB, AP-1 and CREB transcriptional activity in DLD-1 and LS174T cells after glutamine supplementation of H. bilis (MOI 50) infected cells. Cells were transiently transfected with a luciferase reporter plasmid and infected with H. bilis at an MOI of 50. 3mM of L-glutamine (Supplementary Gln) was added in addition to the 2mM already present in the culture medium. H. bilis Δggt infected cells, at MOI of 50 were used as a control. L-glutamine free medium was used to starve the cells of glutamine. Results are expressed as mean of relative luciferase activity to renilla of three independent experiments, normalized to the untreated control. *p<0.05,**p<0.005, ***p<0.0005. Asterisks on top of bars indicate significance relative to untreated control; asterisk on bars indicate significance level between indicated conditions. B) Western blot analysis of p-IκBα and c-Jun protein levels after glutamine supplementation of H. bilis (MOI 50) infected DLD-1 cells after 10 hours of treatment. CREB phosphorylation was investigated in LS174T cells after 10 hours of treatment. One representative blot is shown. (TIF) [file pone.0073160.s003.tif]

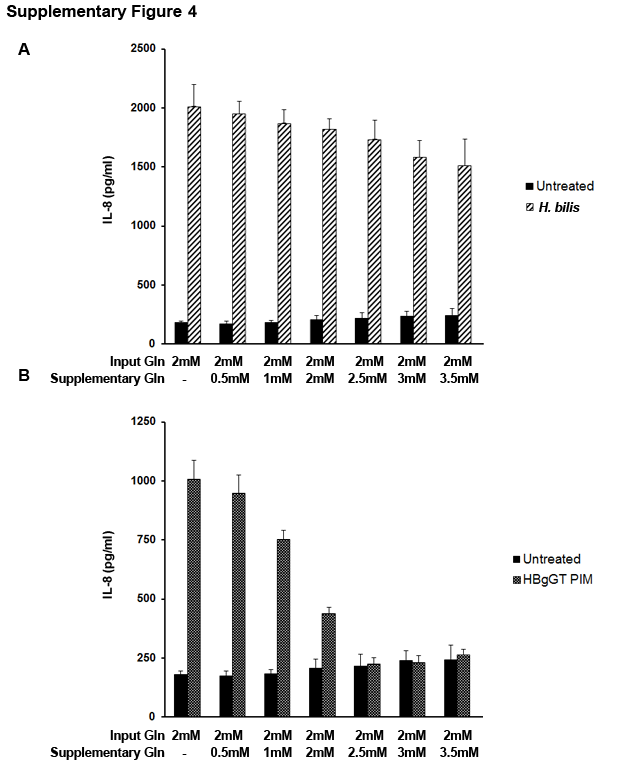

Supplement: Figure S4 — A) IL-8 production in HCT116 cell culture supernatants determined by ELISA in response to increasing supplementary L-glutamine concentrations after 24 hours of H. bilis (MOI 50) infection. Results from two independent experiments conducted in duplicates are shown. B) IL-8 levels after glutamine supplementation of HBgGT PIM treated HCT116 cells at increasing dosage. Supernatants of 24 hour treated cells were collected and IL-8 secretion determined by ELISA. Data from two independent experiments conducted in duplicates are shown. (TIF) [file pone.0073160.s004.tif]
